# VS-FPM: Large-Format, Label-Free Virtual Histopathology Microscopy

**DOI:** 10.34133/bmef.0206

**Published:** 2025-12-02

**Authors:** Christopher Bendkowski, Adam P. Levine, Manuel Rodriguez-Justo, Laurence B. Lovat, Marco Novelli, Michael Shaw

**Affiliations:** ^1^ UCL Hawkes Institute and Department of Computer Science, University College London, London, UK.; ^2^ Research Department of Pathology, University College London, London, UK.; ^3^ Department of Cellular Pathology, University College London Hospitals NHS Foundation Trust, London, UK.; ^4^ Division of Surgery and Interventional Science, University College London, London, UK.; ^5^ HCA Laboratories, London, UK.; ^6^ Department of Chemical and Biological Sciences, National Physical Laboratory, Teddington, UK.

## Abstract

**Objective:** This article describes a new method (VS-FPM) for analysis of unstained tissues based on the application of supervised machine learning to generate brightfield hematoxylin and eosin (H&E) images from phase images recovered using Fourier ptychographic microscopy (FPM). **Impact Statement:** VS-FPM has several advantages for label-free digital pathology. Capture of complex image information simplifies model training and allows post-capture refocusing. FPM images combine high resolution with a large field of view, and the hardware is low-cost and compatible with many existing brightfield microscope systems. **Introduction:** By generating realistic histologically stained images from label-free image data, virtual staining (VS) methods have the potential to streamline clinical workflows, improve image consistency, and enable new ways of visualizing and analyzing histological tissues. **Methods:** We trained a conditional generative adversarial network to translate high-resolution FPM images of unstained tissues to brightfield H&E images and assessed the method using diagnosis of colonic polyps as a test case. **Results:** We found no statistically significant difference between the spatial resolution of FPM images captured at 4× magnification and images from a pathology slide scanner at 20× magnification. Visual assessment and image similarity metrics showed that VS-FPM images of unstained tissues closely resemble images of chemically H&E-stained tissues. However, the spatial resolution of virtual H&E images was approximately 20% lower than equivalent images of chemically stained tissues. Using VS-FPM, board-certified pathologists were able to accurately distinguish normal from dysplastic tissues and derive correct pathological diagnoses. **Conclusion:** VS-FPM is a reliable, accessible VS method that also overcomes many other limitations inherent to histopathology microscopy.

## Introduction

Conventional histopathology is based on the analysis of thin sections of stained tissues using brightfield microscopy. Chemical stains enable visualization of tissue structure, cellular and subcellular organization and morphology, and the distribution of specific proteins for diagnosis, prognosis, and assessment of the efficacy of therapeutic interventions. However, chemical staining adds time to clinical workflows, increases costs, and results in toxic by-products and substantial amounts of wastewater. Differences between reagents and staining protocols increases intra- and inter-laboratory variability, which is of growing importance with the increasing utilization of artificial intelligence based-image analysis [1]. Extensive tissue processing increases the risk of specimen contamination (carry over). Typically, a single stain is applied to each tissue section, and once stained, the same piece of tissue is often not suitable for further assessment using complementary biochemical and molecular analysis. As a result, virtual staining (VS) methods, in which a deep neural network (DNN) is trained to transform label-free image data into an equivalent histologically stained brightfield image, present an attractive alternative to the use of chemical stains in both diagnostic histopathology and research [2].

Since the DNN-based VS concept was first introduced [3], a number of different imaging techniques have been employed for generating contrast from unstained tissues [4], including autofluorescence [3] and fluorescence lifetime microscopy [5], quantitative phase imaging [6], and photoacoustic microscopy and reflected light confocal microscopy [7]. A variety of supervised and unsupervised DNN architectures have been used for the task of translating label-free images into the histologically stained brightfield images required for diagnostic assessment by pathologists [2,4]. Generative adversarial networks (GANs), including conditional [8] and cycle GANs [9], are most commonly employed; however, other methods such as diffusion models have also been used [10].

In this article, we introduce a new method (VS-FPM) for virtual histological staining based on the application of Fourier ptychographic microscopy (FPM) [11] to capture complex images of slide-mounted tissue sections. High-resolution FPM phase images are virtually stained with a DNN trained in a conditional GAN framework using paired FPM phase and amplitude images captured before and after chemical hematoxylin and eosin (H&E) staining. Compared to other label-free imaging methods, FPM offers considerable advantages for digital pathology [12], including experimental simplicity and low hardware cost. By reconstructing high-resolution images from data captured using a low-magnification, low-numerical aperture (NA), objective lens, FPM enables large-format, high-information content imaging of large tissue sections without stage scanning. Recovery of sample amplitude and phase, along with an estimate of the microscopic pupil function [13], allows images to be digitally refocused after capture—during [14] or after reconstruction [15]—for correction of sample tilt and focus errors. Complex images captured before and after chemical staining simplify the pixelwise registration of image data for DNN training compared to other VS methods. The use of light-emitting diode (LED) illumination means that phase images recovered using FPM are free from the interference artifacts that can affect coherent holographic imaging techniques [6].

To test VS-FPM, we built a simple FPM platform using off-the-shelf components and captured images of formalin-fixed paraffin-embedded (FFPE) sections of colonic polyp biopsies before and after H&E staining. Comprising a range of different cell types (including epithelial, stromal, and inflammatory cells) and dysplastic and normal regions, colonic polyps enabled evaluation of the performance of VS-FPM for different tissue structures and cell morphologies. We compare FPM images of chemically H&E-stained (cH&E) tissues to images captured using a state-of-the-art brightfield slide scanning system, virtually H&E-stained (vH&E) FPM phase images to cH&E FPM amplitude images, and vH&E images to brightfield whole slide images (WSIs) of stained tissues, evaluating results using image similarity metrics, empirically estimated spatial resolution, and qualitative diagnostic assessment. Our results demonstrate that VS-FPM is an accessible, clinically valuable method for histological examination of unstained tissues. Finally, we discuss the relative advantages and limitations of VS-FPM compared to other VS methods and wider applications of the technique in research and clinical practice.

## Results

### Comparison of FPM and WSIs for cH&E tissue sections

**Fig. 1.**
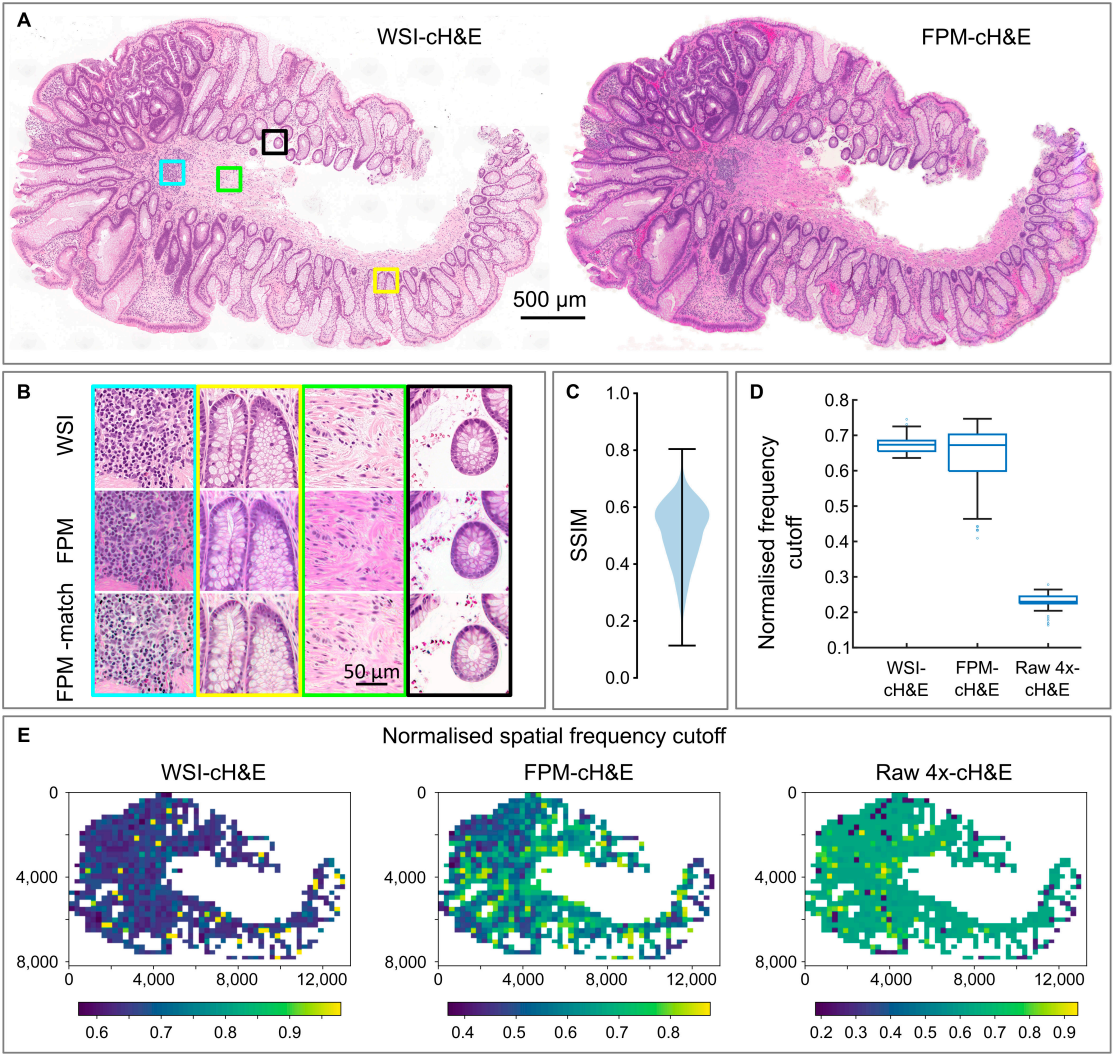
Comparison of WSIs and FPM images of H&E-stained colonic polyps. (A) Representative WSI and FPM amplitude images of an H&E-stained polyp section. (B) Magnified images of the boxed regions in panel (A), showing WSI (top row), FPM image with simple self-white balancing (middle row), and FPM image histogram matched to the WSI (bottom row). (C) Violin plot of structural similarity index between WSI and FPM amplitude images of 18 different polyp sections. (D) Box plots showing the cutoff frequencies for WSI, FPM, and raw 4× images of the section shown in (A) estimated using decorrelation analysis. (E) Heatmaps showing the spatial variation in (min–max normalized) spatial frequency cutoff for images of the tissue section in panel (A).

To compare the imaging performance of FPM (with a 4×/0.16 objective lens) to a brightfield slide scanner (with a 20×/0.75 objective lens), we captured and analyzed images of the same set of 18 H&E-stained polyp sections using both systems. The low magnification of the FPM system meant that a polyp section typically fit within the FPM field of view (FoV), minimizing the need for stage scanning. Figure 1A shows representative overview images of one section from both imaging systems. The shape of the tissue section, the arrangement and morphology of large features such as crypts, and variations in the number density of cell nuclei are clearly visible in both the FPM and WSI results. The magnified regions of interest (RoIs) in Fig. 1B illustrate that detailed features, including the spatial organization of the tissue, cellular morphology, variations in hematoxylin staining, and the detailed structures of crypts, are also clearly resolved in the FPM images. The most striking visual difference between the WSI and FPM results is the color of the images. FPM color images were generated from 3 monochrome images captured sequentially under narrowband illumination from red, green, and blue LEDs, whereas WSIs were captured using a slide scanner with a color camera and white light illumination. As a result, the systems are expected to have very different color gamuts [16] and simple self-white balancing is insufficient to match the color of the subsequent images. The effect of improved color correction/white balancing on the visual appearance of the FPM images is highlighted by the lower row of magnified images (Fig. 1B), in which the reconstructed FPM amplitude image was histogram matched to the WSI result.

The violin plot in Fig. 1C shows the structural similarity index (SSIM) values between the WSI and FPM amplitude images measured for 20,597 patches of 256 × 256 pixels from all 18 H&E-stained polyp sections. The results (mean SSIM of 0.51 ± 0.11) reflect the differences in color and spatial resolution discussed above, as well as WSI and FPM image acquisition (focusing) and reconstruction (stitching and blending) errors. SSIM values tend to vary gradually over a tissue section (Fig. S1A), suggesting that sample tilt, thickness variation, or mounting errors may explain some of the differences. There is no widely accepted SSIM threshold in digital pathology; however, color variations alone can result in substantial differences. For example, one study of color normalization techniques for histological images [17] found SSIM values of 0.7 to 0.8 for images from 2 commercial WSI systems.

**Fig. 2.**
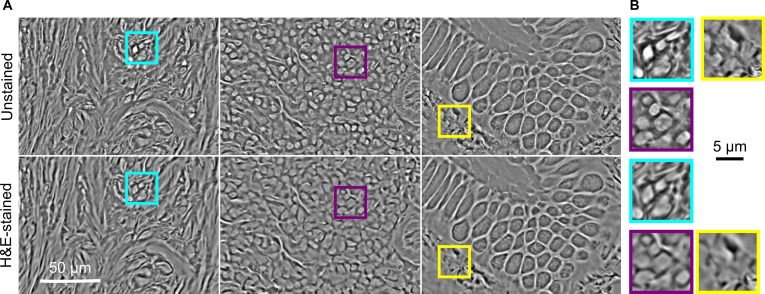
Example FPM phase images of unstained (top) and chemically H&E-stained (bottom) polyp sections. Colored boxes in (A) indicate regions of interest shown at higher magnification in (B).

The nominal spatial resolution of a reconstructed FPM image is typically defined by the synthetic NA of the system $\left({\mathrm{NA}}_{\mathrm{syn}}\right)$, with the cutoff frequency given by $f_{\max}=\lambda /{\mathrm{NA}}_{\mathrm{syn}}$. The NA of the objective lens in the slide scanner was 0.75, similar to the nominal ${\mathrm{NA}}_{\mathrm{syn}}$ (0.7) of the FPM hardware. As such, images from both systems were expected to have similar spatial resolution. However, in practice, the presence of noise and imaging aberrations affect the imaging performance and resolving power. To empirically compare the lateral spatial resolution of the WSIs and FPM images, we used decorrelation analysis to estimate the spatial frequency cutoff for 90 512 × 512 pixel patches from the images shown in Fig. 1A. Analysis of the results using Welch’s *t* test indicated no statistically significant difference (*P* = 0.29) between the mean cutoff frequency for the WSI and FPM image patches—0.67 and 0.68, respectively. The mean cutoff frequency for the 4× brightfield images (computed from a linear sum of the 177 images in the FPM image sequence) was 0.26, indicating that the spatial resolution of the reconstructed amplitude is approximately 2.6 times that of raw image. However, as the cutoff frequency box plots (Fig. 2D) indicate, there was a considerably greater variance (0.095) in measured cutoff frequency for the FPM images than for the WSI (0.024) and 4× brightfield images (0.015). The variation in spatial resolution for the different image types is also shown by the normalized cutoff frequency heatmaps in Fig. 1E—heatmaps for other tissue sections are shown in Fig. S2. The amount of light scattered into the objective lens at darkfield illumination angles is strongly dependent on the local sample properties, specifically local differences in refractive index due to tissue inhomogeneity. Thus, we hypothesize that the greater variance in measured cutoff for the FPM amplitude images is due (at least in part) to differences in the amount of information contained within the darkfield images for each image patch.

### Comparison of cH&E FPM amplitude and virtually H&E-stained FPM phase images

Having established that FPM amplitude images of H&E-stained polyp sections captured using a 4× objective were similar to brightfield WSI images captured at 20×, we next investigated the accuracy with which FPM phase images of unstained tissues could be virtually H&E stained using a conditional GAN. When viewed at low magnification, the reconstructed phase image of a tissue section is visually similar before and after chemical staining (Fig. 2A). However, upon closer inspection (Fig. 2B) image contrast for stained tissues is lower than for unstained tissues, and phase images of stained tissues lack some of the fine details (such as sub-nuclear structure) visible in the unstained equivalents. Although the phase images were sufficiently similar for reliable pixelwise registration of the complex image data captured before and after chemical staining, preliminary tests indicated that the effect of chemical staining on the phase was large enough to markedly degrade the performance of a VS model trained using data from stained sections only.

**Fig. 3.**
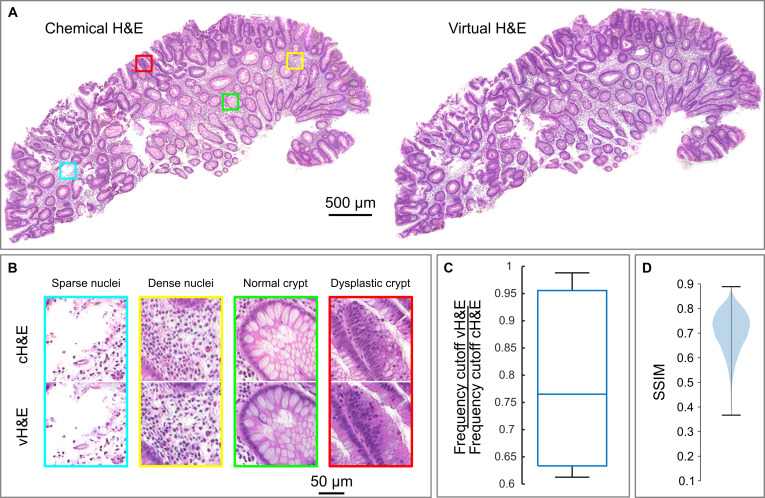
Comparison of chemically (c) H&E-stained FPM amplitude images and virtually (v) H&E-stained FPM phase images. (A) Representative FPM-cH&E and FPM-vH&E images of a colonic polyp section. (B) Magnified images of the boxed regions shown in panel (A), showing features in cH&E and corresponding vH&E images. (C) Box plot showing the relative cutoff frequency for FPM-vH&E images compared to FPM-cH&E images for the images shown in (A). (D) Violin plot of structural similarity index for patches sampled from corresponding cH&E and vH&E images of 6 different polyp sections.

As a result, we trained a conditional GAN-based VS model using pixelwise registered pairs of FPM phase and amplitude patches from 12 polyp sections captured before and after H&E staining, respectively. The generator was then used to virtually stain reconstructed FPM phase images of 6 unstained sections excluded from the training set. Figure 3A shows representative low-magnification images of one of these sections. There is a close visual resemblance between the FPM amplitude image of the H&E-stained section (cH&E image) and the VS-FPM (vH&E image). Viewed at high magnification (Fig. 3B), FPM-vH&E images contain much of the detailed information visible in the FPM-cH&E images. Importantly, normal and dysplastic crypts, characterized by differences in morphology and the number and density of cells and nuclei, can be clearly distinguished in both cH&E and vH&E images. We anticipated that the downsampling half of the U-Net in the VS model would reduce spatial resolution. To quantify this, decorrelation analysis was performed to estimate the spatial frequency cutoff in corresponding FPM-cH&E and FPM-vH&E images, with the result (Fig. 3C) that the mean cutoff frequency in the vH&E images was measured to be 79% of that in cH&E images.

The similarity of FPM-cH&E and FPM-vH&E images was quantified by computing the SSIM for matched images of the 6 polyp sections excluded from the training data (Fig. 3D). The mean SSIM over 3,498 patches was 0.71 ± 0.08, comparable to SSIM values reported in other VS studies based on quantitative phase [6] and autofluorescence imaging [18]. The visual and quantitative similarity between chemically and virtually stained images is particularly striking considering the extensive sample processing (coverslip removal, staining, and re-coverslipping) which took place between acquisition of image data for the unstained and chemically stained tissues.

### Comparison of WSI images of H&E-stained sections and VS-FPM images of unstained sections

**Fig. 4.**
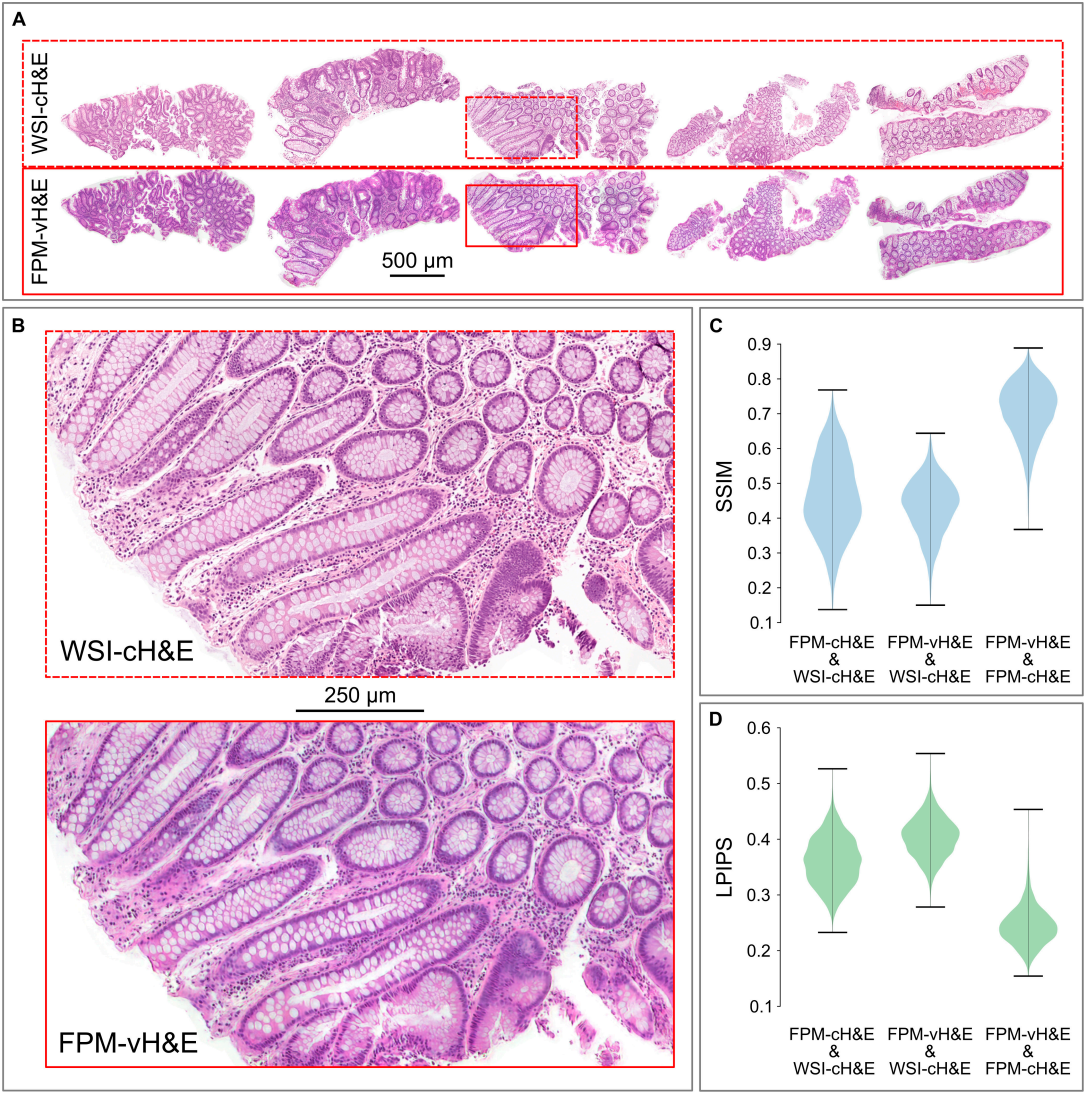
Comparison of WSI images of H&E-stained sections and VS-FPM images of unstained sections. (A) Low-magnification brightfield WSIs (top) and virtually stained FPM phase images for 5 polyp sections. (B) Magnified images of a region of interest from one tissue section. (C) Violin plots showing SSIM and (D) LPIPS values for permutations of H&E-FPM, WSI, and VS-FPM image results. Mean (± standard deviation) values are as follows: SSIM_FPM-cH&E – WSI-cH&E_ = 0.46 ± 0.11, SSIM_VS-FPM – WSI_ = 0.43 ± 0.08, and SSIM_FPM-cH&E –FPM-vH&E_ = 0.71 ± 0.08. LPIPS_FPM-cH&E – WSI-cH&E_ = 0.36 ± 0.04, LIPIPS_FPM-vH&E – WSI-cH&E_ = 0.40 ± 0.04, and LPIPS_FPM-cH&E – FPM-vH&E_ = 0.24 ± 0.03.

As a final test of VS-FPM, we compared images of chemically stained polyp sections captured using a brightfield slide scanner (WSI-cH&E) to FPM-vH&E images of the same sections inferred from phase images captured prior to H&E staining. When viewed at low magnification (Fig. 4A), FPM-vH&E and WSI-cH&E results are similar, with the same tissue structure and large-scale features (including crypts and variations in cell number and density) apparent in both image datasets. To investigate differences in color, we computed CIE Lab* channel histograms for the different image classes (Fig. S3), finding similar distributions for FPM-cH&E and FPM-vH&E, with a mean color difference (Δ*E*) of 8.8. Larger color differences were found between WSI-cH&E and FPM-cH&E and WSI-cH&E and FPM-vH&E images, with mean Δ*E* values of 14.4 and 17.4 respectively. Differences in chromaticity and lightness histograms for the FPM images compared to WSI results are particularly apparent for neutral colors (a* ~ 0, b* ~ 0) and high lightness values (*L* > 90). The compound effects of differences between the FPM and the slide scanner images and FPM-cH&E and FPM-vH&E results, means that differences in structural/morphological features are also apparent when images are viewed at higher magnification (Fig. 4B). These differences are particularly noticeable where nuclei are densely packed (such as in the lower part of Fig. 4B). The violin plots in Fig. 4C and D indicate that FPM-vH&E and FPM-cH&E images are similar with relatively high SSIM and low learned perceptual image patch similarity (LPIPS) [19] values of 0.71 ± 0.08 and 0.21 ± 0.04, respectively. We find markedly larger differences between corresponding FPM-cH&E and WSI-cH&E images (SSIM = 0.46 ± 0.11, LPIPS = 0.35 ± 0.04) and FPM-vH&E and cH&E-WSI images (SSIM = 0.43 ± 0.07, LPIPS = 0.41 ± 0.04). Although Pearson correlation coefficient (PCC) values suggest that FPM-vH&E results are similarly close to both WSI-cH&E and FPM-cH&E (0.73 ± 0.10 and 0.78 ± 0.11, respectively), PSNR and RMSE results show the same trend as for the SSIM and LIPS metrics (Fig. S4). Taken together, these results suggest that differences between FPM-vH&E images and WSI-cH&E images are due primarily to differences between the slide scanner and FPM systems, rather than reflecting a fundamental limitation of inferring vH&E from FPM phase images.

Image difference metrics such as SSIM are widely used to assess differences between images in radiology [20] and digital pathology [21]; however, the values do not always correlate exactly with assessments made by human observers. Further, such metrics only provide a means to compare images, not assess their absolute fidelity. In the case of the present work, our implicit and necessary assumption was that, as the current gold standard for digital pathology, brightfield WSIs from the slide scanner represented a ground truth. However, studies have highlighted variations in images from (nominally similar) brightfield slide scanners that impact measurements of diagnostic features [22]. To further evaluate the VS-FPM method, FPM-cH&E images of 18 polyps and FPM-vH&E images of 6 polyps (excluded from the GAN training set) were reviewed independently by 2 board-certified pathologists who were blinded to the diagnoses. In all cases, both pathologists were easily and accurately able to distinguish normal from dysplastic tissue and derive correct pathological diagnoses from both FPM-cH&E and FPM-vH&E images. Although no formal scoring system was applied, the assessments relied on standard histopathological features such as architectural disorganization, cytological atypia, and abnormal nuclear morphology. These features were consistently, definitively, and concordantly identifiable in FPM images, supporting the practical diagnostic utility of the approach.

## Discussion

We have shown that FPM phase images of unstained colonic polyps can be virtually stained using a conditional GAN to recover brightfield-like H&E images from which reliable and reproducible histological diagnoses can be made. Empirically, FPM amplitude images of chemically stained polyp sections captured using a 4×/0.16 objective lens were found to have a spatial resolution equivalent to images captured using a slide scanner with a 20×/0.75 objective lens. However, vH&E images had a spatial resolution approximately 20% lower than cH&E equivalents, likely due to image downsampling during inference. The use of high-definition GAN architectures [23] may offer a way to minimize this resolution loss and preserve fine features required for some diagnostic applications. At low magnification, visual differences between FPM amplitude and WSI images were due primarily to color variation, and the development of improved color calibration methods for FPM and VS-FPM is one way in which the VS-FPM results could be better matched to WSI data. Although, we note that a lack of color standardization is also an issue in conventional digital pathology [24], and large variations in color have been reported even when imaging samples using nominally similar scanner technologies due to differences in specimen thickness, staining methods, and scanner parameters [25]. In the present work, we trained a VS model using paired FPM phase and amplitude images of unstained and chemically stained tissues. Alternatively, by including an additional step to register WSI-cH&E images to FPM-cH&E amplitude images, a model could instead be trained using registered pairs of WSI-cH&E and FPM phase images. In principle, this may increase the similarity of FPM-vH&E to WSI-cH&E results by accommodating differences between the imaging modalities within the VS model. However, in practice, we found that this additional step introduced registration errors that substantially degraded the training data.

FPM has several features that make it particularly attractive for virtual histological staining. Phase images recovered before and after chemical staining simplify the image registration process required to create paired images for network training. Previous work using phase imaging for VS^6^ required a multistep registration process that involved training a separate neural network for approximate phase to brightfield transformation. Decoupling the microscope FoV from the (diffraction-limited) spatial resolution of the objective lens enables entire tissue sections to be contained within a single FoV, reducing the need for sample scanning. Use of a low-NA objective lens increases the depth of field of the microscope, enabling digital post-image capture refocusing [15] to correct sample tilt and defocusing errors. FPM systems can be adapted to optimize FoV, spatial resolution [26], and image acquisition rate [27] depending on user requirements. A conventional transmitted light microscope can be converted into an FPM system by incorporating an LED array and using a simple microprocessor to synchronize illumination with the microscope camera, meaning the method can be readily adopted in clinical and research laboratories. The relatively low cost and wide availability of LED arrays and microcontrollers [28] also means that there is a low barrier to adoption of the technology in resource-constrained settings, particularly as recovery of the microscopic pupil function enables correction of imaging aberrations associated with other low-cost imaging hardware [29,30].

VS methods comprise 2 core elements: a method for capturing images of unstained tissues and a computational workflow for translating label-free image information into histologically stained images. VS-FPM can be further developed by advancing both of these. We observed marked differences between sample phase images captured before and after chemical staining, likely due to absorption of light by H&E at the wavelength (530 nm) of the green LEDs in our FPM system. Other studies [31] have found that the effect of histological staining on phase images is negligible when using a light source at a wavelength for which there is minimal absorption from the stain. As a result, we anticipate that modifying our setup to include a longer wavelength red LED (~730 nm) could enable the VS network to be trained using data from chemically stained tissues only. In addition to supervised GANs, such as pix2pix, researchers have successfully employed a variety of other DNN architectures, and the optimal method for generating a given histological stain from a given label-free imaging modality remains an open question which requires further study.

Since the technique was first proposed [11], researchers have explored a variety of different FPM hardware configurations and image reconstruction methods to improve the accuracy and spatial resolution of complex FPM images and increase experimental throughput. One fundamental limitation of conventional FPM is the non-uniform phase transfer function, which leads to suppression of low-frequency phase information. That tissue boundaries and cell nuclei are clearly resolved in our FPM phase images may be attributable in part to this effective high-pass filtering, which tends to emphasize the edges of phase objects. In spatially coded FPM [32], a thin non-uniform layer or film in front of the camera sensor enables recovery of low-frequency phase information. For a small increase in experimental and computational complexity, this improves the accuracy of FPM phase images, which may enable corresponding improvements in VS fidelity. Other methods relevant for digital pathology include full FoV FPM reconstruction methods that reduce stitching artifacts associated with patchwise image reconstruction [33] and deep learning [34] and iterative color transfer filtering methods [35] that enable high-resolution color images to be reconstructed from single-channel (monochrome) image data. FPM hardware can be modified to capture more information by detecting perturbations to other properties of the microscopic vector light field. For example, the addition of polarizing elements enables visualization of the birefringence properties of biological specimens [36]. Other (lensless) optical ptychography techniques [37,38] can also capture high-resolution, large-format images suitable for digital pathology. At the cost of increased hardware complexity associated with sample stage scanning these methods offer potential advantages over FPM, particularly in terms of limits to sample thickness and FoV, for VS applications.

Having demonstrated VS-FPM for a specific use case, an obvious question is: can it generalize to other tissue types and stains? Differences in optical path length mean that tissue sections are clearly visible against the slide background and, owing to the lower refractive index of the cell nucleus compared to the cytoplasm [39], nuclei stand out against the surrounding tissue in FPM phase images (Fig. S5). The characteristic ellipsoidal shape of nuclei and the visibility of structural and textural features of the other tissue components provides an explanation for how the VS network is able to apply a virtual hematoxylin stain to nuclei and an eosin counterstain to the other tissue components. Previous studies have demonstrated that quantitative phase images of liver and kidney can be virtually stained for Masson’s trichrome and Jones’ stains and we anticipate that VS-FPM is likely to be effective for virtually staining tissues with these and other special stains that highlight tissue structure. Further, stain-to-stain translation methods [40,41] offer a way to perform virtual immunohistochemical (IHC) staining of unstained tissues via reconstructed vH&E images. Alternatively, by exploiting the absorbance of unstained tissues at shorter wavelengths, deep ultraviolet (DUV) FPM [42] setups can recover high-resolution amplitude images of unstained tissues. Image contrast based on the differential DUV absorbance of endogenous biomolecules may improve VS results when phase images lack the required specificity. Obviating the need for chemical staining has clear benefits for analysis of FFPE tissue sections; however, VS methods also have wider applications in histopathology. The complex field reconstructed using FPM enables post-capture refocusing and reconstruction of extended depth of field images over an axial range corresponding to the depth of field of the objective lens (approximately 20 μm for the system used in this study), indicating that VS-FPM may be suitable for analyzing thicker specimens such as cell smears in cytopathology and sections of fresh, unprocessed tissue in intraoperative histopathology. By increasing the objective NA and the largest illumination angle, high-resolution FPM systems can recover amplitude and phase images with a synthetic NA > 1 [26], for high-resolution diagnostic imaging applications such as karyotyping [43]. Our results, and the inherent flexibility of the method, indicate that VS-FPM can be adapted for a range different applications in histopathology and cytopathology.

## Materials and Methods

### Experimental and technical design

**Fig. 5.**
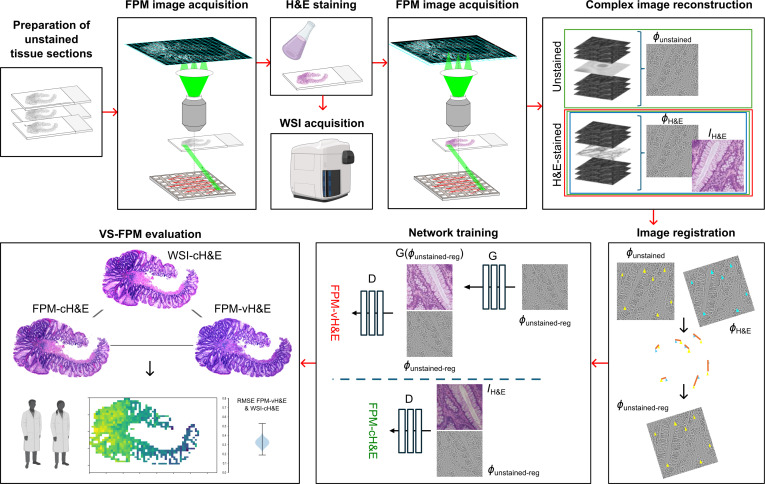
Experimental overview. Sections of slide-mounted colonic polyps biopsies were imaged before and after H&E staining to train a virtual staining model in a GAN framework and evaluate the VS-FPM method.

The experimental workflow used in this study is shown in Fig. 5. Images of unstained sections of colonic polyp biopsies were captured using a custom-built FPM system. After H&E staining, the same sections were imaged using FPM and a brightfield pathology slide scanner. High-resolution complex images were then reconstructed from raw FPM image sequences using iterative phase retrieval. Phase images of unstained tissues were registered to amplitude images of H&E-stained tissues via the corresponding phase images of H&E-stained tissues. Paired image patches were used to train a VS model using a GAN and the results were evaluated using image difference metrics and pathology panel assessment.

### Tissue slide preparation

Anonymized FFPE blocks from colonic polyps (tubular adenomata with low-grade dysplasia) that were surplus to diagnostic requirements were obtained from a clinical histopathology archive. Sections (4 μm thick) were cut using a microtome, mounted on standard glass microscope slides, and deparaffinized. Each section was then sealed with DPX mountant and a coverslip to minimize contamination. After FPM imaging, coverslips were removed by submerging the slides in xylene for 48 h before H&E staining and sealing with a new coverslip.

### Fourier ptychographic microscopy

Image data were captured using a custom-built upright FPM system [44], in which samples were illuminated individually by (red, green, and blue) LEDs in a 22 × 22 array (7 mm pitch, WS212B, WORLDSEMI) controlled via an ESP32-based microcontroller. A 3D printed conical aperture was mounted above the LED array to minimize stray light caused by shallow angle reflections between the lower surface of the glass slide and the top surface of the LED packages. Each raw, monochromatic, FPM dataset comprised 177 images corresponding to sequential illumination from the LEDs within a circle centered on the optical axis. With the LED array at a distance of 81 mm below the top surface of the sample slide, this resulted in reconstructed images with a synthetic NA (${\mathrm{NA}}_{\mathrm{syn}}={\mathrm{NA}}_{\mathrm{obj}}+\sin\theta_{\max}$, where ${\mathrm{NA}}_{\mathrm{obj}}$ is the NA of the objective lens and $\theta_{\max}$ is the largest illumination angle) of approximately 0.7. The imaging pathway consisted of a 4×/0.16 objective lens (UPLSAPO, Olympus) mounted on a piezoelectric focus scanner (P-725.1CDE2, Physik Instrumente) and a tube lens with a focal length of 200 mm (TTL200-A, Thorlabs Inc.), resulting in an effective magnification of 4.44. Images were captured using a large-format scientific CMOS camera (IRIS 15, Photometrics) synchronized to the LED array. This setup provided an FoV of 4.8 mm × 2.8 mm—in many cases sufficient to capture an entire polyp section within a single image. The focus scanner was used for autofocusing at each wavelength before image acquisition by maximizing the normalized variance of images captured under darkfield illumination from a ring of LEDs on the LED array. Microscope slides were mounted on a motorized XY stage (H101A, Prior Scientific) for sample positioning and coarse alignment of images captured before and after H&E staining. Images of unstained tissue sections were captured under illumination by green LEDs (CWL ~ 530 nm) as a compromise between FoV, lateral resolution, image contrast, and sampling in Fourier space. Color images of H&E-stained tissue sections were captured under sequential illumination by red (CWL ~ 629 nm), green, and blue (CWL ~ 475 nm) LEDs. Brightfield images were captured with an exposure time $\left(T_{\mathrm{BF}}\right)$ of 2 ms. To improve the signal-to-noise ratio, the exposure time ($T_{n}$) for each darkfield image was adjusted in proportion to the squared distance $d_{n}$ of the illuminating LED from the optical axis, $T_{n}\propto T_{\mathrm{BF}}\cdot {d_{n}}^{2}$. The intensity I of the captured images was then scaled down accordingly, $I_{n}=I_{\mathrm{raw}}\cdot T_{\mathrm{BF}}/T_{n}$.

Captured FPM sequences (177 5,056 × 2,960 pixel images) were processed using an iterative Gauss–Newton phase retrieval algorithm [45] to reconstruct high-resolution complex images. For reliability and computational simplicity, images were reconstructed in patches of 243 × 243 pixels and upsampled by a factor of 3 to accommodate the additional high-resolution information. Scalar offsets and gradients between phase image patches were corrected by normalizing each patch by first subtracting the mean phase and setting the standard deviation to unity. A Gaussian blur (with a kernel size 31 pixels) was then applied to a copy of the patch, and this blurred patch was subtracted from the normalized patch. Background regions in the corrected phase images were thereby set to ~0. To correct for intensity variations in reconstructed amplitude images of chemically stained sections, the high-resolution amplitude patch and the corresponding brightfield low-resolution patch (formed from the sum of the brightfield images captured in the FPM stack) were histogram matched. Following reconstruction and correction, amplitude and phase patches were stitched using the Grid/Collection stitching ImageJ plugin [46] to reform a full high-resolution FoV (15,142 × 8,867 pixels). Radial and tangential distortion in the high-resolution images were then corrected using calibrated camera parameters to give the flat image necessary for image registration before and after staining. To correct for lateral chromatic offsets, red, green, and blue amplitude images of H&E-stained sections were aligned using an affine transform derived from matched SIFT [47] features. The aligned RGB images were then self-white balanced using a manually defined background RoI.

### Whole slide imaging

WSIs of H&E-stained polyps were captured using a NanoZoomer S360MD (Hamamatsu Photonics K. K.) in 40× mode with a pixel size of 230 nm. The images were stored in a pyramidal format and the (downsampled) 20× images were used for comparison to FPM image results for stained and unstained sections.

### Virtual H&E staining

A VS network was trained using pixelwise registered pairs of FPM phase images of unstained tissues and the corresponding RGB FPM amplitude images after H&E staining. Absorption of light by H&E modified the local refractive index of the stained tissue sections and had the effect of blurring out some of the fine features visible in phase images of unstained sections (Fig. 2). However, the overall similarity of FPM phase images before and after chemical staining enabled reliable image registration of complex images. Matched SIFT features [47] were used to find corresponding key points in the unstained and stained phase images, before RANSAC [48] was applied to find a perspective transform. As the VS network required small patches to train on, the high-resolution matched sets of unstained phase and stained phase and amplitude images were divided into patches of 256 × 256 pixels. Each patch was then assessed using mutual information of the phase image histograms to determine if the match was of sufficient quality to be included in the training dataset. A cutoff value of 0.4 was used to exclude outlier patches, which included those containing reconstruction artifacts or those corresponding to tissue-free background parts of the slide.

VS was performed using the Pix2Pix cGAN architecture [8] with an objective defined as

$$G^{*}=\text{arg }\underset{G}{\text{min }}\underset{D}{\text{max}}\;L_{\text{cGAN}}\left(G,D\right)+{\lambda L}_{L1}\left(G\right),$$ (1)

where $L_{\text{cGAN}}\left(G,D\right)$ is the objective of the cGAN as defined in the original Pix2Pix article [8], ${\lambda L}_{L1}\left(G\right)$ is the L1 loss of the generator, and λ = 100. The training phase comprised 100 epochs and used a dataset of 17,199 matched pairs of unstained phase and H&E-stained RGB amplitude image patches sampled from 12 different tissue sections. The dataset was augmented during training by applying random jitter and mirroring. During inference, the memory limitations of the GPU (RTX 3090, NVIDIA) restricted the image size that could be virtually stained; thus, the full high-resolution (15,142 × 8,867 pixels) FoVs were divided into 1,024 × 1,024 pixel patches, which were then stitched together using the Grid/Collection stitching ImageJ plugin [46] to create a virtually stained FoV. When required, FoVs were stitched using the BigStitcher ImageJ plugin [49] to produce an image of a whole slide.

### Image evaluation

Differences between brightfield WSIs, FPM amplitude images of H&E-stained tissues, and VS-FPM images of unstained tissues were quantified using SSIM, PSNR, RMSE, and PCC metrics computed from grayscale images (Y) converted from the RGB images using

Y=0.299·R+0.587·G+0.114·B. (2)

For a pair of images x and y, each comprising M·N pixels and with a maximum pixel value of R, these were defined as

$$\text{SSIM}\left(x,y\right)=\frac{\left(2\mu_{x}\mu_{y}+C_{1}\right)\left(2\sigma_{\mathrm{xy}}+C_{2}\right)}{\left(\mu_{x}^{2}+\mu_{y}^{2}+C_{1}\right)\left(\sigma_{x}^{2}+\sigma_{y}^{2}+C_{2}\right)}$$ (3)

(where $\mu_{x/y}$ and $\sigma_{x/y}$ are the mean and standard deviation of image x/y, $\sigma_{\mathrm{xy}}$ is the covariance of images x and y, and $C_{1}$ and $C_{2}$ are small constants used to avoid instability),

$$\text{RMSE}\left(x,y\right)=\sqrt{\frac{\sum_{M,N}{\left(x\left(m,n\right)-y\left(m,n\right)\right)}^{2}}{M\cdot N}},$$ (4)

$$\text{PSNR}\left(x,y\right)=10{\text{log}}_{10}\frac{R^{2}}{{\text{RMSE}}^{2}},$$ (5)

$$\mathrm{PCC}\left(x,y\right)=\frac{\sigma_{\mathrm{xy}}}{\sigma_{x}\sigma_{y}}.$$ (6)

Differences between color images were calculated using the LPIPS metric described by Zhang et al. [19] using LPIPS v0.1.4 (available at https://github.com/richzhang/PerceptualSimilarity) with the default pretrained AlexNet. LPIPS calculates similarity by comparing the activations of a predefined network for 2 image patches, giving a result between 0 and 1, where lower values indicate higher similarity. CIE Lab* histograms were calculated by converting the RGB images to CIE Lab* using the function cvtColor in OpenCV version 4.10.

Image resolution was quantified using the spatial frequency cutoff estimated using image decorrelation analysis, as described by Descloux et al. [50]. Briefly, a decorrelation function was computed from the cross-correlation of the 2D Fourier transform (FT) of the image normalized by its amplitude and filtered by a series of circular binary masks with the unnormalized, unmasked 2D FT of the image. Decorrelation functions were then computed for a series of high-pass filtered images, and the cutoff was defined as the highest frequency at which a local maximum was detected in the decorrelation function.

## Data Availability

The image data presented in this article are available at https://doi.org/10.6084/m9.figshare.29086454.
