# Supplementary material for: VS-FPM: Large-Format, Label-Free Virtual Histopathology Microscopy
Source: BME Front. 2025 Dec 2;6:0206. doi: 10.34133/bmef.0206 (PMC12669476; doi:10.34133/bmef.0206)
Supplement: Supplementary 1 — Figs. S1 to S5 [file bmef.0206.f1.zip › SUPPLEMENTARY MATERIALS.docx]

SUPPLEMENTARY MATERIALS


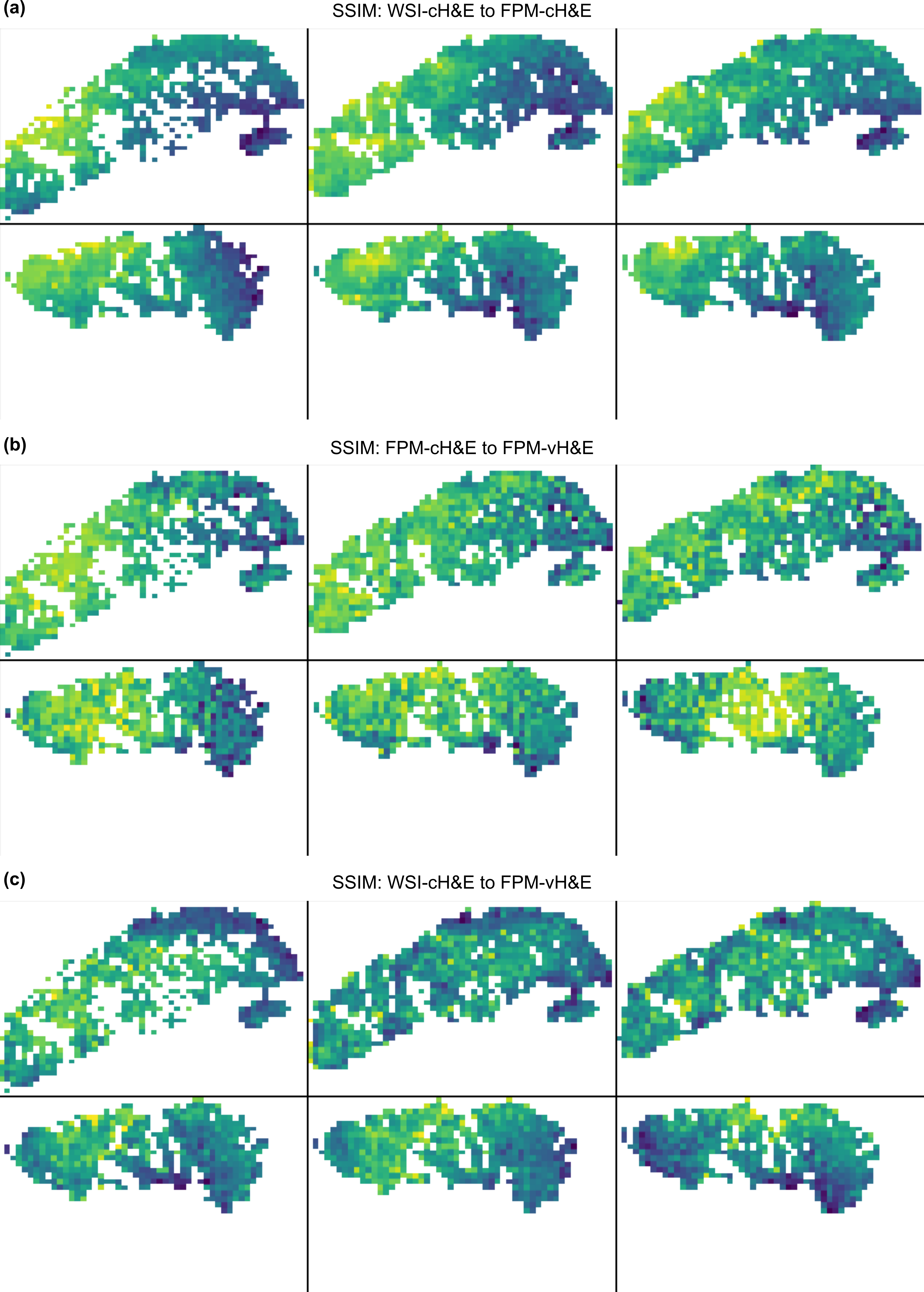


**Figure S1. Heatmaps showing spatial variation in structural similarity of paired images for six polyp sections.** (a) SSIM heatmaps for WSI against FPM amplitude images for H&E-stained sections. (b) SSIM heatmaps for FPM amplitude image of chemically H&E-stained tissue against virtually H&E-stained FPM phase images. (c) SSIM heatmaps for WSI images of chemically H&E-stained tissue against virtually H&E-stained FPM phase images. Heatmaps min-max normalized for each modality pairing.


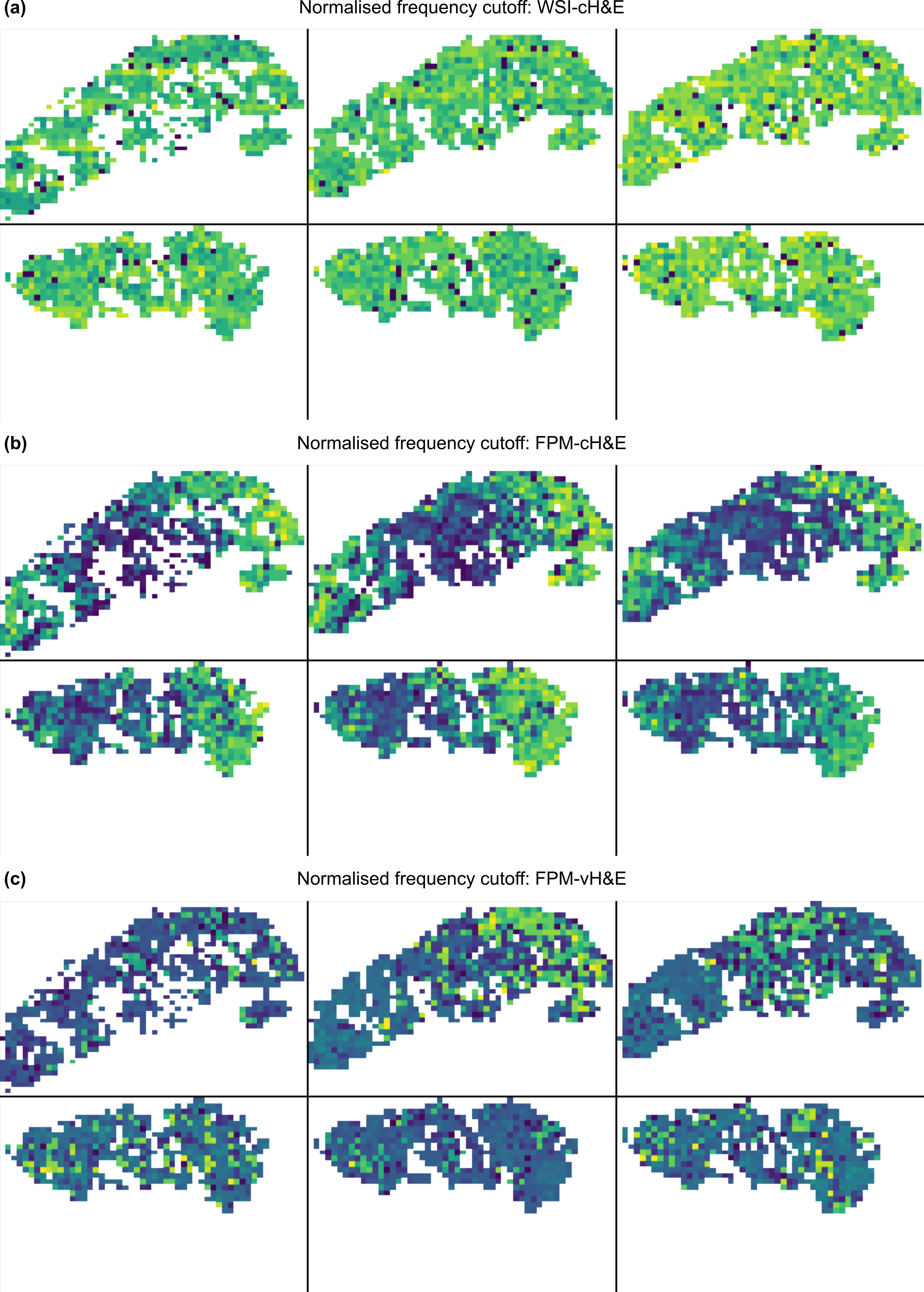


**Figure S2. Heatmaps showing spatial variation in normalized spatial frequency cutoff.** (a) WSI and (b) FPM amplitude images of six chemically H&E-stained polyp tissue sections, and (c) virtually H&E-stained FPM phase images for the same sections prior to chemical staining. Heatmaps min-max normalized for each modality.


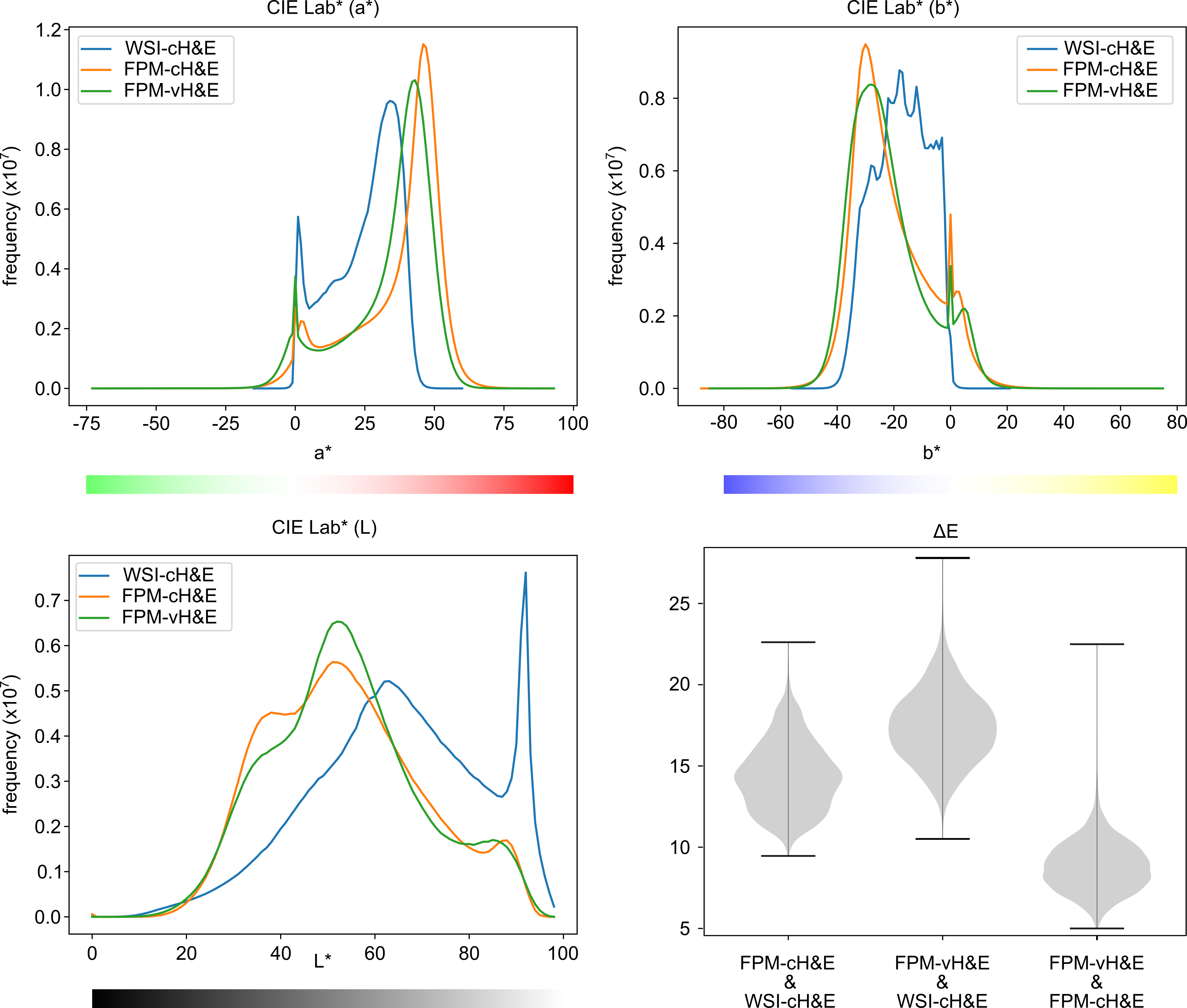


**Figure S3. CIE Lab* chrominance histograms for WSI-cH&E (blue), FPM-cH&E (orange) and FPM-cH&E (green) images.** Data corresponds to 3498 image patches from six different polyp sections. Mean (± standard deviation) colour differences are: ΔE_FPM-cH&E – WSI-cH&E_ = 14.3 ± 2.1, ΔE_FPM-vH&E – WSI-cH&E_ = 17.4 ± 2.2 and ΔE_FPM-cH&E – FPM-vH&E_ = 8.8 ± 1.6.


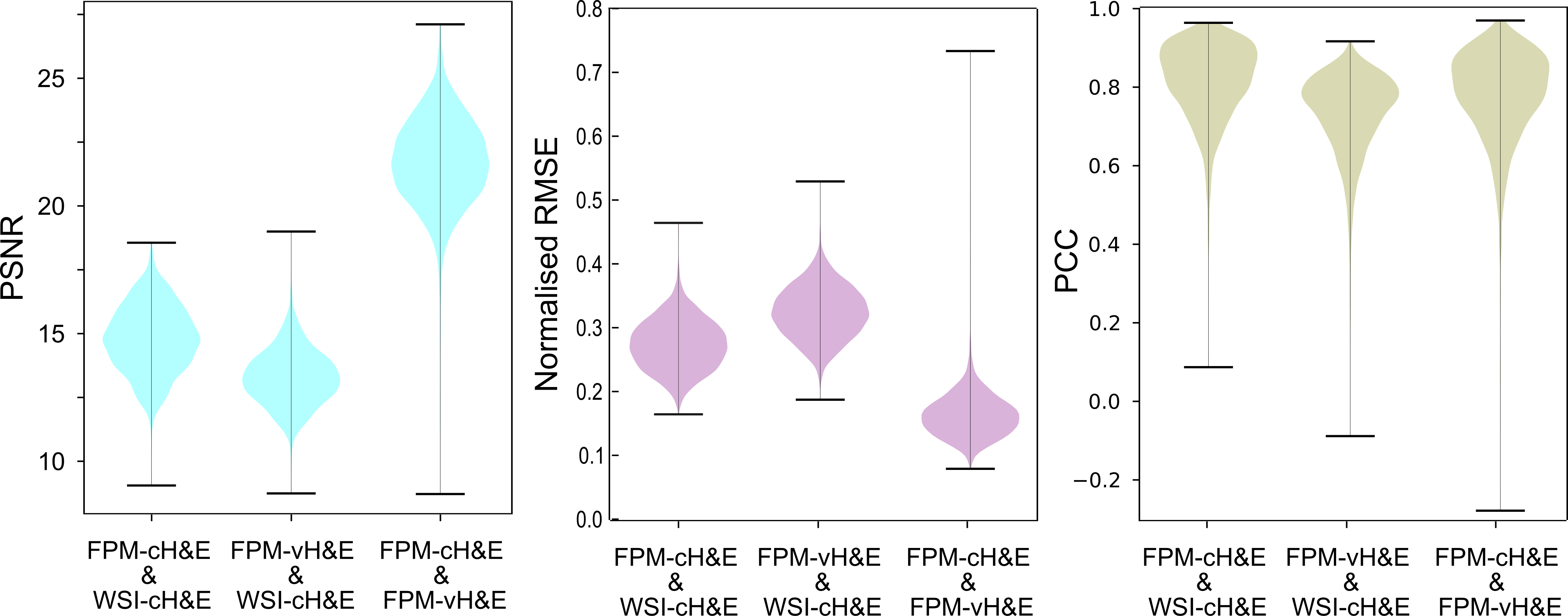


**Figure S4. Peak signal-to-noise (PSNR), normalized root mean square error (RMSE) and Pearson correlation coefficient (PCC) image difference metrics for WSI, and FPM images of chemically-stained sections and virtually-stained sections**. Data corresponds to 3498 image patches from six different polyp sections.


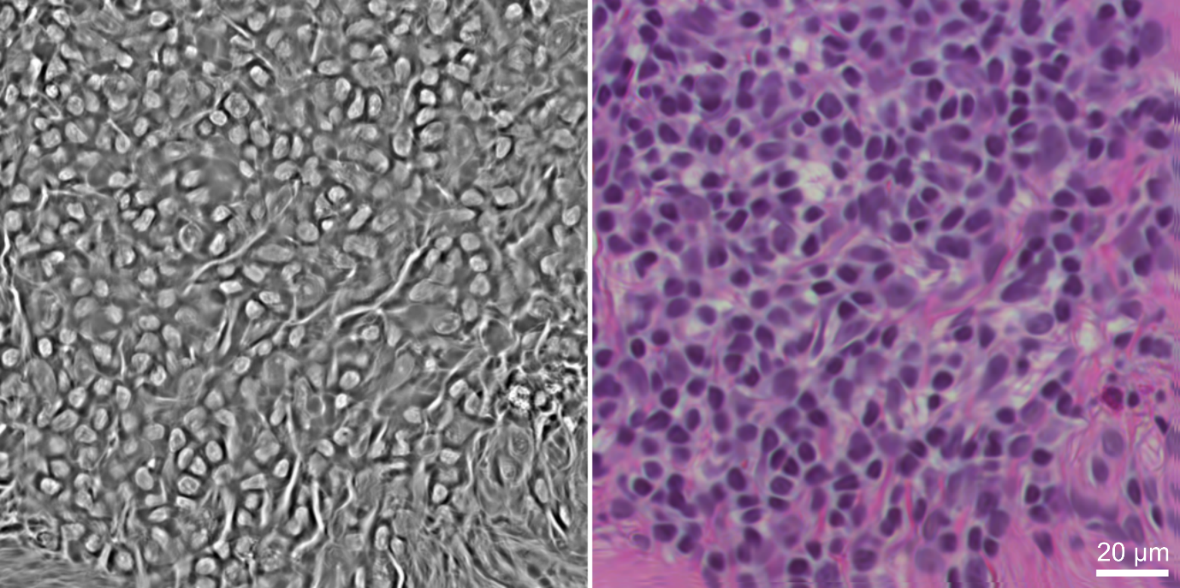


**Figure S5. Representative FPM phase image (left) of an unstained colonic polyp and corresponding FPM amplitude image (right) after chemical H&E staining.** In the phase image cell nuclei are visible as bright quasi-ellipsoidal blobs on a grey background.
