# Supplementary figures and images for: VS-FPM: Large-Format, Label-Free Virtual Histopathology Microscopy
Source: BME Front. 2025 Dec 2;6:0206. doi: 10.34133/bmef.0206 (PMC12669476; doi:10.34133/bmef.0206)

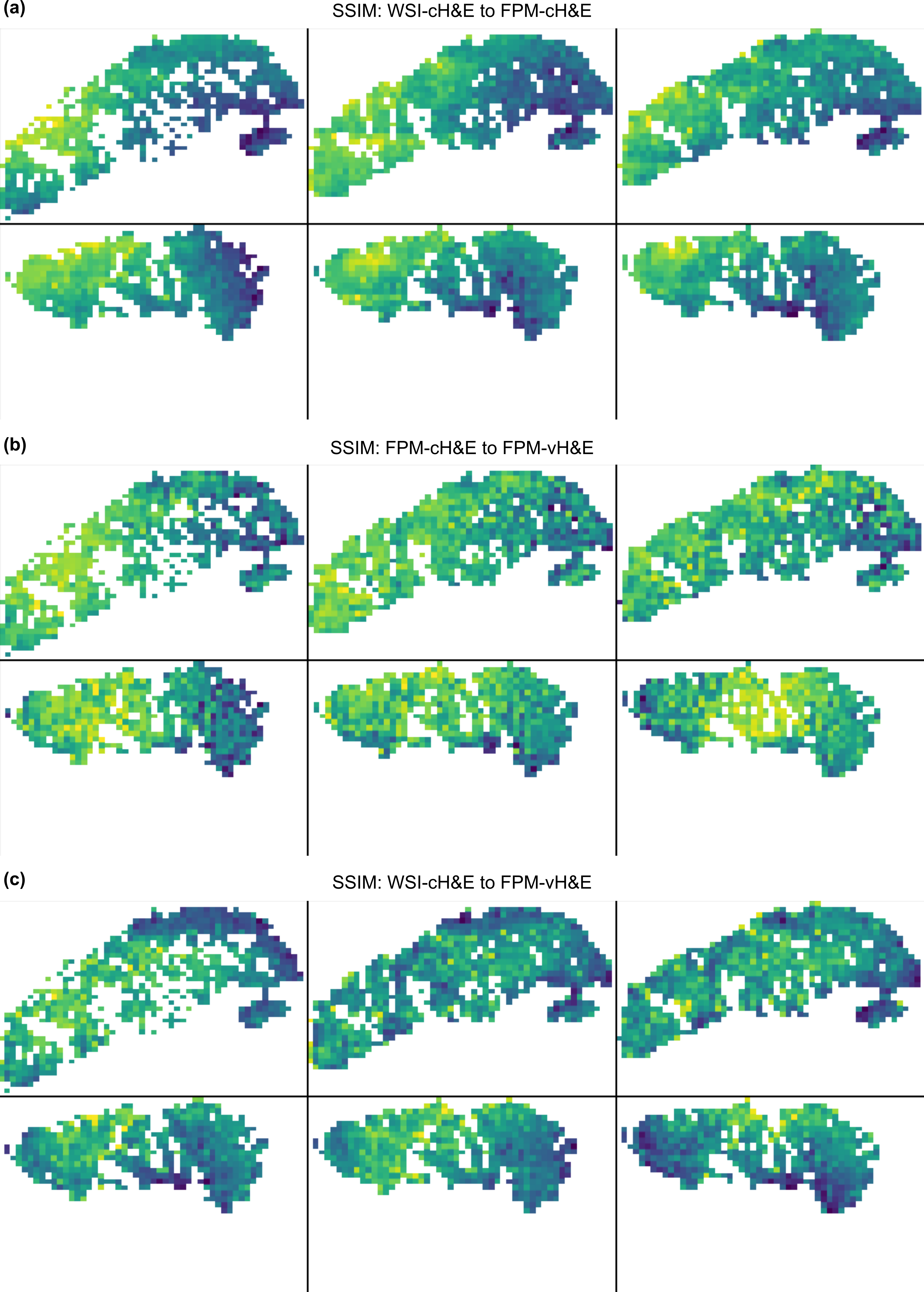

Supplement: Supplementary 1 — Figs. S1 to S5 [file bmef.0206.f1.zip › FigS1.png]

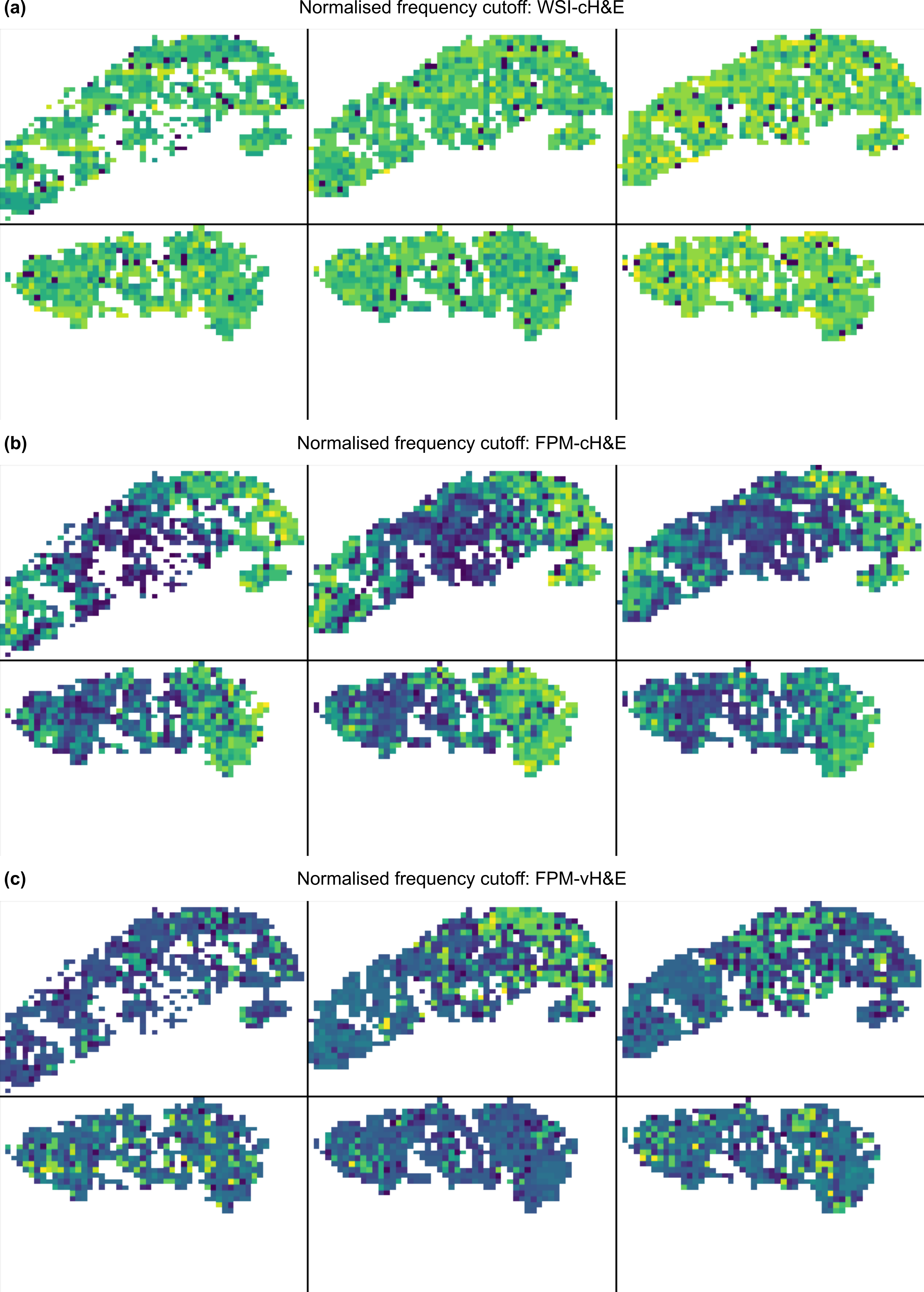

Supplement: Supplementary 1 — Figs. S1 to S5 [file bmef.0206.f1.zip › FigS2.png]

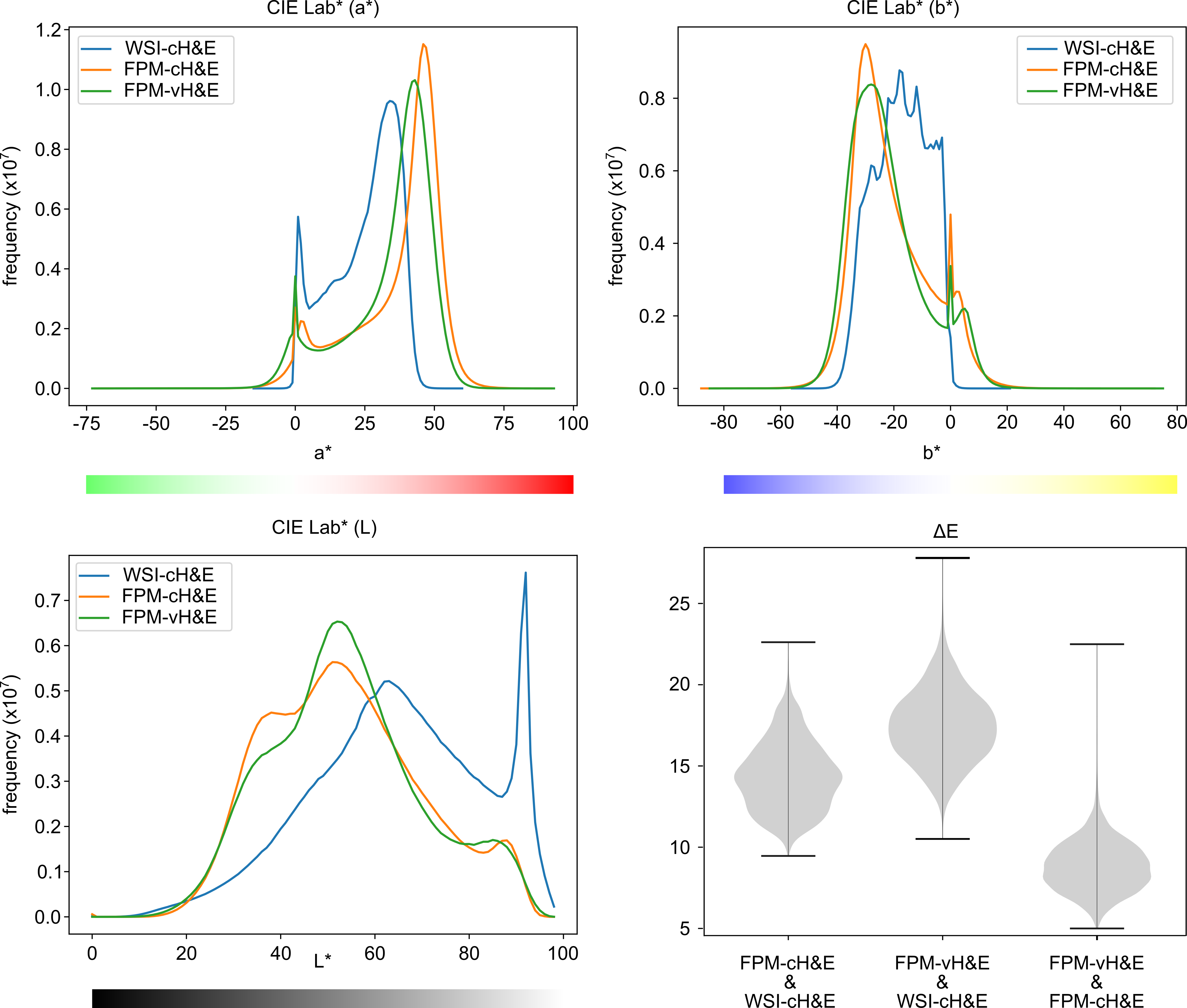

Supplement: Supplementary 1 — Figs. S1 to S5 [file bmef.0206.f1.zip › FigS3.png]

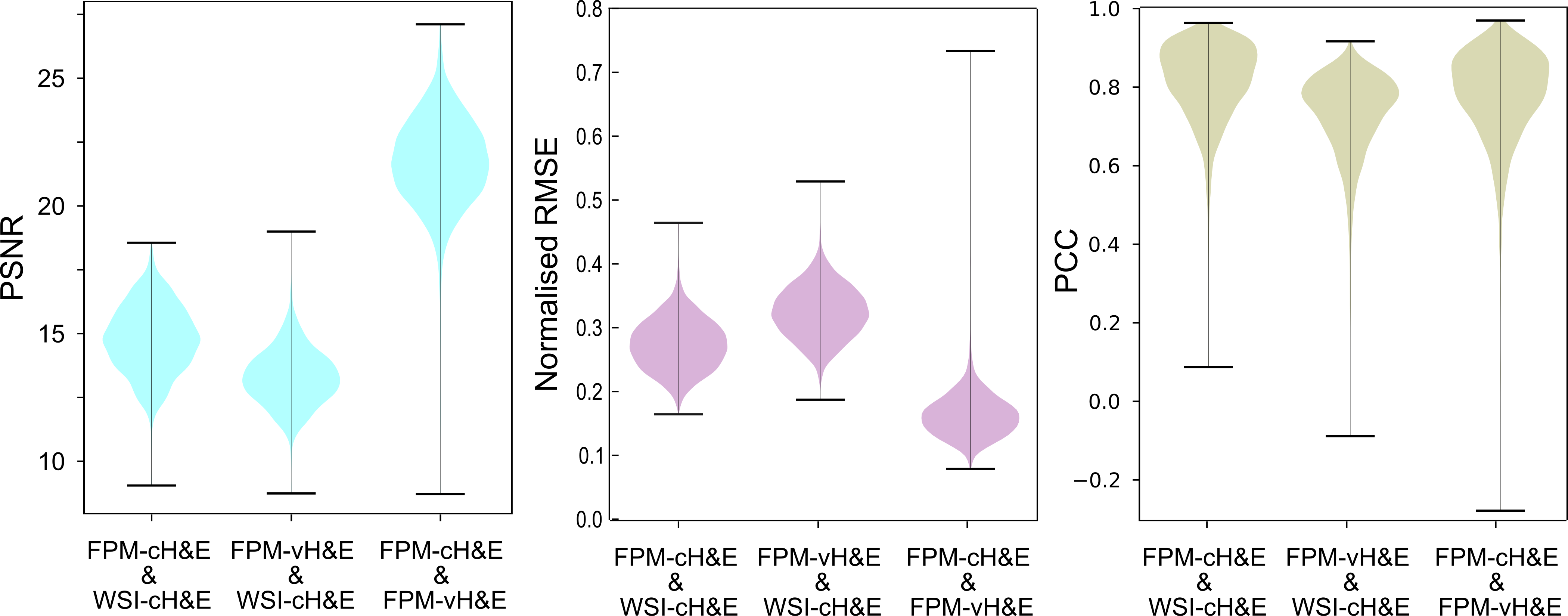

Supplement: Supplementary 1 — Figs. S1 to S5 [file bmef.0206.f1.zip › FigS4.png]

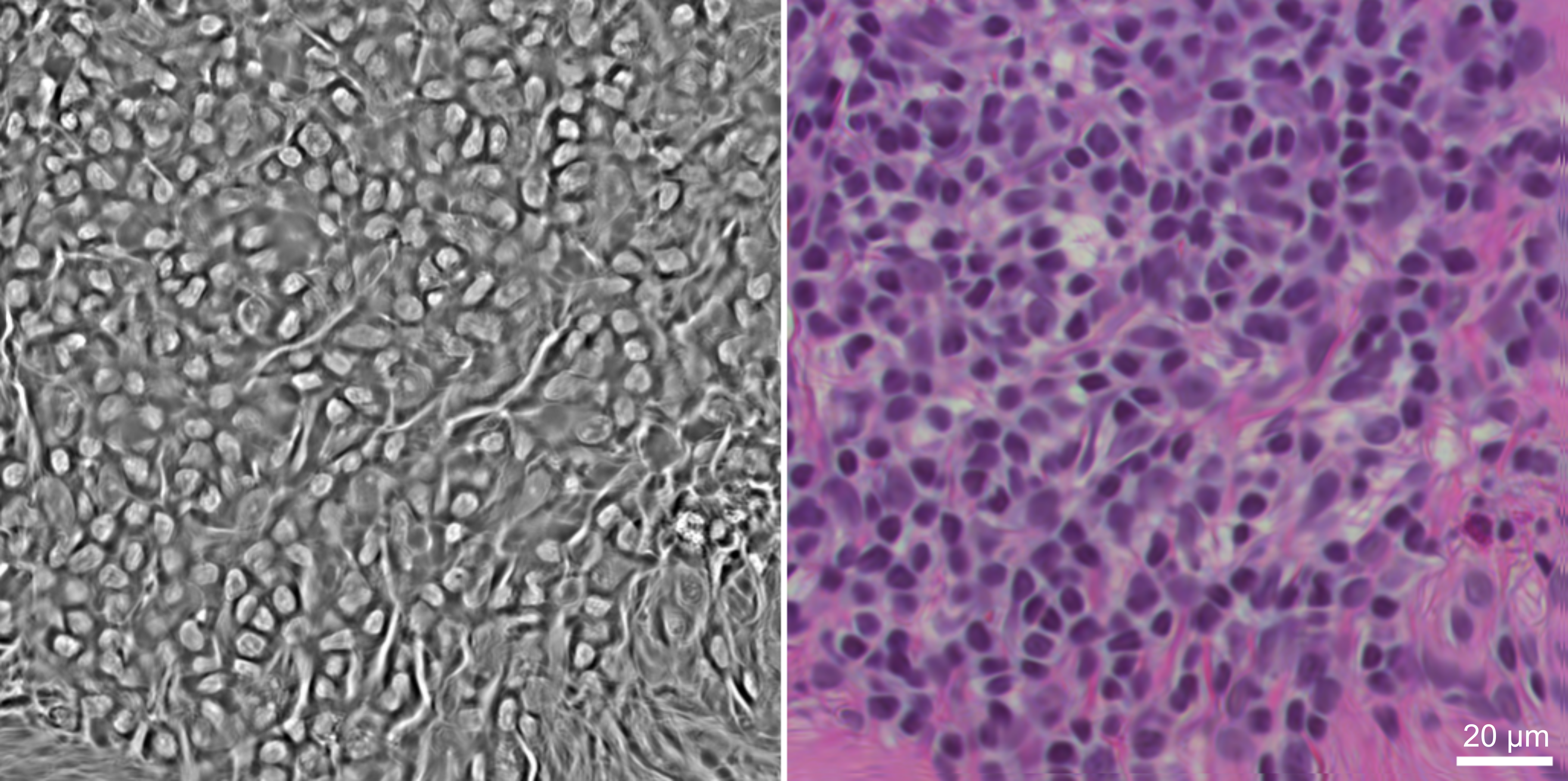

Supplement: Supplementary 1 — Figs. S1 to S5 [file bmef.0206.f1.zip › FigS5.png]
